# The challenges and opportunities of conducting a clinical trial in a low resource setting: The case of the Cameroon mobile phone SMS (CAMPS) trial, an investigator initiated trial

**DOI:** 10.1186/1745-6215-12-145

**Published:** 2011-06-09

**Authors:** Lawrence Mbuagbaw, Lehana Thabane, Pierre Ongolo-Zogo, Trudie Lang

**Affiliations:** 1 Centre for the Development of Best Practices in Health (CDBPH), Yaoundé Central Hospital, Henri Dunant Avenue, Messa, PO Box 87, Yaoundé, Cameroon; 2 Department of Clinical Epidemiology and Biostatistics, McMaster University, Hamilton, ON, Canada; 3 Biostatistics Unit, Father Sean O'Sullivan Research Centre, St Joseph's Healthcare, Hamilton, ON, Canada; 4 Global Health Clinical Trials Programme, Centre for Vaccinology and Tropical Medicine, University of Oxford, Churchill Hospital, UK

## Abstract

Conducting clinical trials in developing countries often presents significant ethical, organisational, cultural and infrastructural challenges to researchers, pharmaceutical companies, sponsors and regulatory bodies. Globally, these regions are under-represented in research, yet this population stands to gain more from research in these settings as the burdens on health are greater than those in developed resourceful countries. However, developing countries also offer an attractive setting for clinical trials because they often have larger treatment naive populations with higher incidence rates of disease and more advanced stages. These factors can present a reduction in costs and time required to recruit patients. So, balance needs to be found where research can be encouraged and supported in order to bring maximum public health benefits to these communities. The difficulties with such trials arise from problems with obtaining valid informed consent, ethical compensation mechanisms for extremely poor populations, poor health infrastructure and considerable socio-economic and cultural divides. Ethical concerns with trials in developing countries have received attention, even though many other non-ethical issues may arise. Local investigator initiated trials also face a variety of difficulties that have not been adequately reported in literature. This paper uses the example of the Cameroon Mobile Phone SMS trial to describe in detail, the specific difficulties encountered in an investigator-initiated trial in a developing country. It highlights administrative, ethical, financial and staff related issues, proposes solutions and gives a list of additional documentation to ease the organisational process.

## Introduction

Randomized clinical trials are the cornerstone of evidence-based decision making and are considered the 'Gold Standard' for clinical research. In recent years a huge scientific revolution towards evidence-informed health care decision-making has boosted the clinical trial industry [1]. The recognition of systematic reviews (which mostly use randomized trials as units of analysis) as important tools in health care decision making has also given a silent nod towards the growth of the industry. The Cochrane collaboration is one of the largest producers of systematic reviews [2].

Even though the clinical trial industry is growing, a simple search by location on the clinical trials website, clinicaltrials.gov [3] will reveal 24 trials in Cameroon as opposed to 7662 in Canada or 50896 in the United States of America (USA). On the World Health Organization (WHO) International Clinical Trials registry Platform (ICTRP), the numbers are similar [4]. These differences reflect the differences in experience in all the parties involved; regulatory bodies, researchers and participants. Additional differences arise from culture, health infrastructure and socioeconomic divides [5]. In low-resource settings, structures such as the Data Safety and Monitoring Board (DSMB), Community Advisory Boards (CAB), Regulatory Authorities (RA) and Institutional Review Boards (IRB) that play key roles in monitoring and approval of trials may be non-existent, non- functional or lack the skills to critically appraise a research protocol. In Cameroon the National Ethics Committee (NEC) plays all these roles. In consequence, trial capacity is lacking, and research centres in low resource settings are unable to lead large scale independent research projects [6].

For the purposes of accrued external validity, low costs, faster recruitment and in some cases, reduced set-up times, commercial clinical trials are increasingly being conducted in middle income countries like China and India [5]. It is estimated that a trial in China or India could cost somewhere between a third to half of what it would cost in the United States. Also, the incidence rates of some diseases may be too low for a large scale trial in a developed country [7]. Commercial (for-profit) research initiatives will understandably aim for the least costly settings, and will invest primarily in drugs and technologies, as they need to make profits. Whilst there is of course a need for a robust supply chain of new drugs and vaccines for all settings, we also need to support research that helps improve the way we use existing therapies and manage illnesses. These disease management trials benefit the local populations and respond to the countries' priorities [5]. They are highly effective mechanisms for bringing about improvements in disease outcomes. Examples include finding new uses for antibiotics, or evaluating how to improve nurse training.

There are relatively few commercial trials in developing countries and a lack of suitably trained and resourced centres to conduct non-commercial research. Therefore the situation is that little research is performed in the low income countries with the highest disease burden.

The ideal solution for this research dilemma would be for local investigators to lead the initiation and conduct of nationally relevant trials, as they will be more in tune with the socio-cultural context, the disease burden, national priorities and infrastructural challenges.

The literature on the conduct of clinical trials in low resource settings mostly covers ethical issues, and the need to avoid double standards or the difficulties in obtaining meaningful informed consent in places were community consent applies. They address post-trial access to care and compensatory mechanisms with particular reference to middle-income Asian countries such as India and China [8-12]There is a lack of data on trial methods and operational challenges in low- income countries globally. There is a need for researchers based in developing countries to work together and share their experiences, methods and resources. There are some initiatives beginning to meet this need [6,13], and here we provide some of our experiences.

The main aim of this paper is to discuss the issues surrounding the conduct of the Cameroon Mobile Phone SMS (CAMPS) trial [14], an investigator-initiated and designed trial in a low-resource setting. We do not intend to be critical of any health system or administrative procedures in low resource setting countries, rather we hope to share our experiences from the CAMPS trial to shed light on key challenges that researchers may encounter in similar settings and how to address them. Some of the issues raised may also apply to multi-centre trials with some sites in low resource settings, adding to the relevance of sharing research experiences with researchers worldwide.

Here we describe the CAMPS trial in the context of its setting, discuss the administrative, ethical and financial issues related to the CAMPS trial and how the human resources were managed. We also provide a list of documents which can improve the management of a trial and the quality of the data collected. We have made these documents available for others to use on the Global Health Trials website (http://ght.globalhealthehub.org/articles/).

## Discussion

### Type of study

CAMPS is an investigator-initiated, randomized controlled trial of motivational mobile phone text messages versus routine care to improve adherence to Anti-Retroviral Therapy (ART) among people living with Human Immune Deficiency Virus (HIV) over 6 months. It is a single-blinded trial involving 198 participants divided into two groups. Participants were randomized to receive either the intervention (weekly motivational mobile phone text messages and usual care) or usual care alone. The CAMPS trial [14] is the first mobile phone technology trial in Cameroon. It is also the first registered trial in the country on technological aids to adherence for ART, which we present as a pragmatic example of a locally-led disease management trial.

### Study setting

Situated in West Africa, Cameroon is a resource stricken country with an adult HIV prevalence of 5.1% [15]. The unique study site, the Yaoundé Central Hospital (YCH) Accredited Treatment Centre (ATC) has been used for observational studies, but hardly for clinical trials. It is located in the capital city, Yaoundé. The YCH is a referral hospital with a staff of about 800, including 95 doctors [16]. The ACT provides care for more than 6500 people living with HIV.

### Ethical approval and Administrative issues

#### Ethical approval

The application for ethical clearance was straight-forward, as the National Ethics Committee (NEC) already had a list of requirements to be submitted with the application. The list can be sent by email upon request. A document processing fee must be paid based on the source of the funding.

However, owing to the fact that Cameroon is a bilingual country, some documents, notably the consent form, information booklet, data collection form and investigator brochure had to be submitted in both languages. Using only English and French is far cheaper, than having to translate into all the local languages. Cameroon has 286 local languages[17].

Second, the NEC was unfamiliar with certain standardized reporting tools, namely the Consolidated Standards for Reporting Trials (CONSORT) flow diagram [18] and requested that it be removed from the protocol. Review was completed within a month.

Finally, we were clarified on the legal age of consent in Cameroon, which is 21 years as opposed to 18 years in many other countries, thus redefining our concept of 'children' and modifying our inclusion criteria.

Many papers have identified the ethical challenges of running a trial in a developing country and the need for protecting the developing countries participating in international clinical trials [5,9-11,19]. With drug trials, adverse effects are the main risk involved and can usually be identified directly, sometimes as secondary outcomes. How do you define the risk of receiving a text message? We identified one concern and that was the possibility of disclosure of privacy and potential disclosure of HIV status. Our trial was designed to protect privacy and data. However, we needed to plan for accidental disclosure. If this happened, how could this be managed and what appropriate compensation should be offered? This issue is context specific, especially in a country where there is still some lingering stigma associated with HIV [20]. In other similar trials, loss of privacy was not a deterrent to the intervention [21]. Such implementation difficulties may be encountered frequently, especially since the International Conference on Harmonisation of Technical Requirements for Registration of Pharmaceuticals for Human Use - Good Clinical Practice (ICH-GCP) guidelines[22] offer little guidance for non-drug, non-industry interventions [6,23,24].

Post trial access to care was a challenge and this is a difficult area for many groups planning research in settings such as ours. We discussed this carefully and concluded that it could not be guaranteed without ensuring that the intervention was effective, not unreasonably expensive, could be performed on a large scale and that there was a legal framework established with the mobile operators to ensure anonymity of the participants.

#### Administrative issues

Administrative bottle necks can seriously impede the ability of researchers to set up studies, so much so that delays can make the trial meaningless or out of date by the time the study is allowed to start. This is a serious issue and so it is unfortunate that these aspects are often overlooked in discussions around what impedes research. These governance and organisational aspects are fundamentally important, yet it is difficult to obtain funding, training and support in this area for trial staff. Each modification in the protocol, say for example adding one more interviewer, required a change in the budget. These changes, no matter how slight were difficult to run through administration.

The finances provided for the running of the trial had to pass through the normal financial procedures of YCH and the Centre for the Development of Best Practices in Health (CDBPH). The later is a research-dedicated body while the former is not, however the former (YCH) being hierarchically superior to the latter is responsible for the approval of all purchases and expenditures, and has the right to reject expenses they deem unnecessary. This left little room for modification of the budget, despite the constant need to adapt the protocol due to scientific counsel, ethical recommendations and miscellaneous needs. A second difficulty was the fact that the finances were disbursed in two bi-annual instalments. The YCH required us to submit a complete budget, but within the limits of the account balance. In other words we were not allowed to budget beyond what we had at hand. We found ourselves juggling two budgets, one covering the first instalment and the other covering their entire sum allocated for the project.

### Financial issues

#### Cost of running a trial

Compared to developed countries, the cost of running a trial is significantly cheaper in developing countries. There are many reasons for this, including lower salary and overhead costs and that less time is required to enrol participants [5,7]. The compensatory mechanisms used in this trial, which represent a considerable portion of the budget, are addressed in detail below.

#### Participant compensation

As can be expected, the NEC and the trial research group did not agree with financial compensation for participants. The main reasons advanced were that this practice would hamper future research with less funding, may induce participants into trying to satisfy the researchers and also causes patients to come for unscheduled visits if they will get some transport money. The NEC suggested that we need not specify in the information booklet that there was no financial compensation. We gave them preferential treatment by reducing their waiting times before they could see their doctors. This seemed to be the most acceptable and fairest method of compensation as we were helping them to regain time lost during the interviews.

#### Interviewer compensation

Interviewer salaries were open to more discussion. Some of them had experience with data collection and felt they should be compensated per filled questionnaire. The interviewers and the principal investigator agreed that the workload would be evaluated during the testing of the questionnaire. Bearing in mind the workload involved, the distraction from their regular duties, the need above all for quality data, from motivated interviewers and the difficulty in finding people interested in research we considered a higher financial compensation level. On the other hand, we could not provide compensations which would compete with their usual salaries, as this could be a disincentive to work. We were also limited by budget constraints. Lastly their compensation would also depend on the modalities involved in payment. It was initially unclear whether to pay them per questionnaire filled (would we consider a questionnaire to be baseline data for one patient, or a complete set of baseline, interim and final data?), per month (there were some months during which the interviewers had no planned activities) or a lump sum per data collection period (a salary for baseline, interim and final data collection). We finally decided to pay them lump sums per data collection period, provide communication airtime, and to establish contracts stipulating the number of patients to be enrolled and undisclosed bonuses for promptness and collection of high quality data. Despite this apparently attractive compensation scheme, their salary criterion (fifty patients enrolled per interviewer) was similar to the compensation for recruiting one patient into a foreign sponsored multi-centre trial [25]. A top-up salary payment for staff working in trials in developing countries is a challenging issue for researchers seeking to set up independent trials in these regions. On one hand it is quite understandable that healthcare workers on relatively low salaries should be compensated for carrying tasks beyond their normal roles. However these costs for trials can add up and make them prohibitively expensive. This area needs some discussion between institutions where research is conducted and ministries of health, as a solution could encourage more locally run research that benefits local public health goals.

### Team of investigators and study staff issues

Our project staff comprised a principal investigator (PI), a study site coordinator (SSC), a supervisor and a research group made up of public health physicians, specialists in research synthesis, anthropologists and health economists. We had substantial support from renowned clinical trial experts and a biostatistician from the McMaster University in Canada. We found that an administrative officer was a critical component of the team. He was responsible for ensuring that all documents were translated and submitted on time, organized meetings and trainings for interviewers, drafted all the legal and administrative documents (applications to the YCH and NEC, contracts with interviewers, disbursement of funds, and provision of equipment) managed the budget, and archived all trial related activities. It is critical to mention here that there is a special need for administrative letters to have the right formatting and content without which simple processes can be delayed for months. We also sought guidance, advice and resources from the Global Health Trials website. This platform has created a community of clinical researchers from developing countries who support research through sharing experience and resources.

In a resource constrained setting, human resources (including highly qualified research personnel) are just one of the limited resources. Drawing interviewers from the already limited pool of health workers is the only way to get staff that have some knowledge on the content of the research, are familiar with patient communication and may benefit from the results of well conducted research. Despite a previous training session, our interviewers had difficulties with filling the questionnaire and required repeated explanations on the concept and importance of research techniques like randomization and blinding, and tools like the visual analogue scale. Table 1 summarises the difficulties that may be encountered when organising a trial in a low resource setting and possible ways to address the issues.

**Table 1:** List of difficulties that may be encountered and potential solutions

Difficulties that may be encountered	Potential solutions
Regulatory bodies with different standards	Loco- regional recognition of international standards Adaptation of international standards to local realities

Administrative bottle necks	Creation of research managing bodies within the administration

Financial bottle necks	Budgets should be drawn after extensive ground work

Multiple languages	The local languages should be considered from the conception stages

Patient compensation options	Ethical, feasible options should be employed after consulting with patient associations, other researchers and health personnel

Interviewer compensation modalities	All the options should be discussed openly with the interviewers during the preliminary stages and contracts established

Inadequate interviewer competence	Training and capacity building

Different ages for legal consent	Country specific age of legal consent must be taken into account when drafting the protocol

No dedicated administrative officer	An experienced administrative officer should be hired

### Additional documentation

Each review body typically provides a list of documents required to accompany an application for ethical approval. Table 2 is list of other useful documents that may ease the research process. These additional logs and registers permitted us to collect additional and relevant data that could not be put in the questionnaire, ensure quality control, provide timely feedback to interviewers and also to reduce losses to follow up. These documents complement the ICH-GCP list of essential documents [22], and we have placed them on the Global Health Trials website.

**Table 2:** Additional documents to enhance the quality of trials in low resource settings

Document	Contents	Use
**Interviewer contracts**	Names of interviewer, duration of recruitment period, roles and responsibilities of the investigators and interviewers including number of participants to be enrolled	Clearly defines roles and responsibilities of the concerned parties. Sets individual targets for interviewers

**Recruitment log**	Dates, number of forms filled, interviewer names, problems encountered, refusals and non-eligible subjects	Easily exploitable enrolment data, quality control of data, a good feedback mechanism

**Trial contact list**	Names, functions and phone numbers of everybody involved in the trial	Handy contact list, permits real time communication with interviewers, coaching and encouragement

**Interviewer follow-up form**	Names of participants, dates of enrolment, date of next visit	Essential for interviewers to track patients and prepare for follow-up visits

**Participant feedback log**	Dates, times, contact addresses and content of feedback from participants	Provides ongoing monitoring of intervention Useful data for providing a context for later findings

Trial registration is another important component of the trial process. Registering trials in international registries ensures that the data is made available to the public and limits the possibility of publication bias and selective reporting. It enhances decision making and prevents duplication of efforts. The revised Declaration of Helsinki emphasizes that all clinical trials must be registered in a public data base even before the recruitment of the first subject [26]. This again can be challenging for independent researchers as the process has to be completed by a sponsor. It is often a problem to identify which of the organisations involved can and will act as a sponsor. The sponsor is required to provide trial indemnity insurance.

Finally, trialists are recommended to use uniform reporting criteria like the CONSORT statement to ensure that trial protocols adhere to high design and methodological standards [18,27].

### Current initiatives

Support structures for clinical trials exist in the USA, United Kingdom (UK), Canada and other developed countries. They assist non-commercial trialists, and provide guidance to ensure that their trials are legal and compliant. They also make the regulations more straightforward and less daunting [6]. Capacity-building in clinical trials is still needed in most developing countries. The creation of clinical trial sites in low resource settings based on national research needs and the availability of competent or potentially trainable staff can improve the research culture and promote centres of excellence from which other local researchers can copy good practices. The African Malaria Network Trust (AMANET) and the Gates Malaria Partnership (GMP) are good examples of how capacity strengthening can promote high quality research [28,29]. Partnerships with the high income countries like the European and Developing Countries Clinical Trials Partnership (EDCTP) that promote investigator driven research and concentrate on the major killer diseases, while supporting regulatory and ethics activities may be the way forward [30]. The Global Health Clinical Trials platform is beginning to meet this gap by providing an online professional network for clinical trialists. The network provides a good discussion forum for research staff in resource-limited settings to share their problems and exchange ideas for solutions [13].

## Conclusions and recommendations

Investigator-initiated trials may face similar difficulties with trials sponsored by international bodies. These difficulties will be related to handling the regulatory bodies, administrative and financial bottlenecks, multiple languages, patient compensation options, interviewer compensation modalities, and additional documents required to ease the collection of data.

Based on the current literature and our experience with the CAMPS trial, we recommend training and capacity building for regulatory authorities.

Additionally, independent support networks for clinical trials are necessary to ensure that specific challenges are addressed, tools are provided and protocols are respected.

Delays due to financial and administrative hurdles can be sorted out by channelling funds directly to the point of expenditure. Inclusion of local investigators at the conception and design stages of a trial may help to reduce the likelihood of unforeseen expenditures. Financial audits can also help to safeguard accountability, but should not be an impediment to the research process.

Possible language barriers need to be taken into account as part of the planning. Such plans would need to include the testing and verification of translations for accuracy.

Preparedness and adaptability are essential ingredients for successful conduct of clinical trials in any setting, especially resource poor settings. Having an administrative officer dedicated to handling administrative issues related to the trial can be an invaluable asset. This would also allow the scientist to devote more time to the scientific aspects of the trial. This requirement is necessary when the administrative requirements may represent a substantial hindrance to the progress of the trial.

Multisite trials should have national coordinators selected before the onset of the trial who will participate in the conception of the trial. Investigator-initiated pragmatic trials are arguably the way forward for clinical research in developing countries[31]. Local participants can play an important role in planning trials to ensure that protocols are culturally sensitive and beneficial [32]. Trials should also target the specific medical needs of the country as perceived by the country [33]. It is equally important that the profession of Clinical Trial Scientist be recognized as a viable career path for researchers in developing countries [6].

## List of abbreviations

ACT: Accredited Treatment Centre; AMANET: African Malaria Network Trust; ART: Anti-Retroviral Therapy; CAB: Community Advisory Boards; CAMPS: Cameroon Mobile Phone SMS; CIHR: Canadian Institutes of Health Research; CONSORT: Consolidated Standards for Reporting Trials; CDBPH: Centre for the Development of Best Practices in Health; CTN: Canadian HIV Trials Network; DSMB: Data and Safety Monitoring Boards; EDCTP: European and Developing Countries Clinical Trials Partnership; GCP: Good Clinical Practice; GMP: Gates Malaria Partnership; HIV: Human Immunodeficiency Virus; ICH: International Conference of Harmonisation; ICTRP: International Clinical Trials Registry Platform; IRB: Institutional Review Board; NEC: National Ethics Committee; PI: Principal Investigator; RA: Regulatory Authorities; SSC: Study Site Coordinator; UK: United Kingdom; USA: United States of America; WHO: World Health Organisation; YCH: Yaoundé Central Hospital.

## Competing interests

The authors declare that they have no competing interests.

## Authors' contributions

LM conceived of the paper. LT, POZ and TL helped to draft and review several versions of manuscript. All authors read and approved the final manuscript.
